# Supplementary material for: Cixutumumab reveals a critical role for IGF-1 in adipose and hepatic tissue remodelling during the development of diet-induced obesity
Source: Adipocyte. 2022 Jun 23;11(1):366–78. doi: 10.1080/21623945.2022.2089394 (PMC9235901; doi:10.1080/21623945.2022.2089394)
Supplement: Supplemental Material [file KADI_A_2089394_SM2687.zip › 224363545 Adipocyte R1.docx]

**Cixutumumab reveals a critical role for IGF-1 in adipose and hepatic tissue remodelling during the development of diet-induced obesity**

^1^Helen Imrie*, ^1^Hema Viswambharan*, ^1^Natalie J Haywood, ^1^Katherine I Bridge, ^1^Nadira Y Yuldasheva, ^1^Stacey Galloway, ^1^Katie J Simmons, ^1^Richard M Cubbon, ^1^Piruthivi Sukumar, ^1^Nicole T Watt, ^1^Laeticia Lichtenstein, ^2^Judy I Wyatt, ^3^Hiromi Kudo, ^3^Robert Goldin, ^1^Baptiste Rode, ^1^Stephen B Wheatcroft, ^1^Mark T Kearney.

^1^ Leeds Institute for Cardiovascular and Metabolic Medicine, University of Leeds, United Kingdom. ^2^ Department of Pathology Leeds Teaching Hospitals NHS Trust. ^3^ Department of Metabolism, Digestion and Reproduction, Imperial College, London.

**Abbreviated title:** IGF-1 receptor deletion and obesity

* These authors contributed equally

Address for Correspondence:

Professor Mark Kearney,

The LIGHT Laboratories,

University of Leeds,

Clarendon Way,

LS2 9JT Leeds,

United Kingdom.

Email: [m.t.kearney@leeds.ac.uk](mailto:m.t.kearney@leeds.ac.uk)

Tel: +441133437764

**Abstract**

High fat diet (HFD) induced obesity leads to perturbation in the storage function of white adipose tissue (WAT) resulting in deposition of lipids in tissues ill-equipped to deal with this challenge. The role of insulin like growth factor-1 (IGF-1) in the systemic and organ specific responses to HFD is unclear.

Using cixutumumab, a monoclonal antibody that internalises and degrades cell surface IGF-1 receptors (IGF-1R), leaving insulin receptor expression unchanged we aimed to establish the role of IGF-1R in the response to a HFD.

Mice treated with cixutumumab fed standard chow developed mild hyperinsulinemia with no change in WAT. When challenged by HFD mice treated with cixutumumab had reduced weight gain, reduced WAT expansion and reduced hepatic lipid vacuole formation. In HFD fed mice cixutumumab led to reduced levels of genes encoding proteins important in fatty acid metabolism in WAT and liver. Cixutumumab protected against blunting of insulin-stimulated phosphorylation of Akt in liver of HFD fed mice.

These data reveal an important role for IGF-1R in the WAT and hepatic response to short-term nutrient excess. IGF-1R inhibition during HFD leads to a lipodystrophic phenotype with a failure of WAT lipid storage and protection from HFD induced hepatic insulin resistance.

**Key words**: adipocyte, insulin‐like growth factor (IGF), lipodystrophy, obesity, adipose tissue, browning, triglyceride.

**Introduction**

Obesity secondary to excess intake of lipid is a multisystem disorder associated with disruption of signalling pathways critical to efficient energy utilisation and storage ^1^. A pathophysiological hallmark of obesity is insulin resistance; often defined as impaired ability of insulin to promote glucose uptake in muscle and fat and inhibit gluconeogenesis in the liver ^2, 3^. Insulin may act in synergy with insulin like growth factor-1 (IGF-1) to coordinate responses to nutrient availability. Support for this possibility is provided by the fact that IGF-1 and insulin diverged during evolution from a single common IGF-1/insulin precursor which linked nutrient intake and growth ^4^. Upon nutrient abundance, this precursor was released, stimulating cellular anabolism and tissue growth ^5^. The role of IGF-1 in regulating metabolism in mammals, and its effect on different tissues under different nutritional circumstances, is incompletely explored. While illuminating, studies employing mice with genetic depletion of IGF-1R have limitations and the recent demonstration that the insulin receptor (IR) and IGF-1R can impact on expression of multiple imprinted genes and microRNAs ^6^, highlights the need to examine the role of IGF-1R using complementary approaches to germline deletion. Here, we describe the effect of short-term disruption of IGF-1 signalling using an IGF-1R specific monoclonal antibody to systemically knockdown IGF-1R in mice receiving either a standard chow diet or a high-fat high-calorie diet (HFD). Our data identify the IGF-1R as a critical regulator of white (WAT) and hepatocyte remodelling when challenged by calorie excess.

**Results**

**Pharmacological whole-body reduction of IGF-1R in mice receiving chow diet leads to hyperinsulinemia and insulin resistance**

To investigate the effect of systemically reducing IGF-1R using a non-genetic approach, we treated C57/Bl6J mice with the monoclonal antibody cixutumumab (A12) (Supplementary Figure 1A), which induces internalisation and degradation of IGF-1R, or an isotype control (IC) antibody (kind gifts from ImClone). On a standard chow diet, cixutumumab treated mice had reduced IGF-1R expression (Supplementary Figure 1B) but unaltered IR expression (Supplementary Figure 1C) in liver, skeletal muscle and WAT. Body mass (Figure 1A), heart, kidney and aorta weight (Figure 1B), epididymal fat mass (Figure 1C), white adipocyte area (Figure 1D), and BAT mass (Figure 1E), were similar in cixutumumab and isotype control treated mice. In glucose tolerance tests, cixutumumab-treated mice demonstrated a similar increase in blood glucose as isotype control-treated mice (Figure 1F), whereas in insulin tolerance tests, cixutumumab-treated mice demonstrated reduced insulin sensitivity compared to isotype control-treated mice (Figure 1G). Serum leptin concentrations were similar in cixutumumab-treated mice compared to isotype control treated mice. Serum insulin and IGF-1 concentrations were significantly higher in cixutumumab-treated mice compared to isotype control treated mice (Table 1). There was no difference in liver mass (Figure 1H), hepatic triglyceride content (Figure 1I) or hepatic cholesterol content (Figure 1J) in cixutumumab-treated mice compared to isotype control-treated mice.

**Pharmacological reduction of IGF-1R in mice receiving high-fat diet leads to reduced weight gain, blunted WAT expansion and reduced hepatic lipid vacuole formation**

We, next investigated cixutumumab treatment in the setting of positive energy balance. On HFD, we confirmed cixutumumab-treated mice had reduced IGF-1R expression in liver, skeletal muscle and WAT (Supplementary Figure 1D), but unaltered IR expression (Supplementary 1E**).** Body weight gain (Figure 2A), final total body weight (Supplementary Figure 2A-C) in cixutumumab treated mice, epididymal fat depot mass (Figure 2C), and mean adipocyte area in epididymal and subcutaneous WAT depots (Figure 2E,F) were all reduced in cixutumumab treated mice. Heart, kidney and aorta weight (Figure 2B) were similar in cixutumumab and isotype control treated mice, as was BAT mass (Figure 2D). Lipid content of BAT (Figure 2G), was increased in cixutumumab treated mice. In glucose tolerance tests, cixutumumab treated mice demonstrated a similar increase in blood glucose to isotype control-treated mice (Figure 2H), whereas in insulin tolerance tests, cixutumumab-treated mice demonstrated a tendency to reduced insulin sensitivity compared to isotype control treated mice (Figure 2I). Fasting plasma glucose concentration was significantly increased in chow-fed cixutumumab treated mice compared to the isotype control (Supplementary Figure 2D-E), while fasting plasma glucose levels in HFD fed mice treated with cixutumumab demonstrated no difference. Circulating concentrations of insulin, free fatty acids, triglycerides and leptin were similar in both groups and IGF-1 were elevated in cixutumumab treated mice. Fasting glucose levels in both groups and IGF-1 were elevated in cixutumumab treated mice (Supplementary Figure 3A, B).

**Pharmacological reduction of IGF-1R in mice receiving high-fat diet leads to increased food intake and increased urinary and faecal energy loss**

Cixutumumab treated mice receiving HFD demonstrated increased food intake (Figure 3A), but unaltered water intake (Figure 3B). There was a tendency towards increased urine output (Figure 3C), and faecal output was greater (Figure 3D), in cixutumumab treated mice compared to isotype control treated mice. Daily energy lost in urine (Figure 3E) and lipid lost in faeces (Figure 3F) were greater in cixutumumab treated mice compared to isotype control-treated mice.

**Pharmacological reduction of IGF-1R in mice receiving high-fat diet differentially alters gene expression in WAT and BAT**

Gene expression of *Igf1r, Tnfα, and Lep* was similar in WAT of cixutumumab treated mice receiving HFD, compared to isotype control treated mice (Figure 3G). Gene expression of *Ucp-1, Vegf, Igf-1r, Prdm16, Cidea, Lep, Tnfα*, in interscapular BAT in both high fat and chow-fed groups, were also comparable in cixutumumab treated mice and isotype control treated mice (Figure 3H&I and Supplementary Figure 4A-B**).** In subcutaneous WAT, expression of *Pparγ*, was significantly increased in cixutumumab-treated mice (Figure 3J), but not epididymal WAT (Figure 4A). We also examined expression of genes encoding sterol regulatory element-binding protein 1 (*Srebp-1*)**,** fatty acid synthase (*Fas*), microsomal triglyceride transfer protein (MTTP), HMG-CoA reductase (HMGCR*)* and Acetyl-CoA carboxylase 1 (*Acc*) in BAT, subcutaneous WAT and epididymal WAT. In mice fed a HFD cixutumumab had no effect on these genes in BAT (Figure 4B). In contrast to this in subcutaneous WAT, cixutumumab reduced expression of *Acc, Mttp* and *Hmgcr* mRNAs (Figure 4C). In epididymal WAT cixutumumab reduced expression of the gene encoding *Mttp* (Figure 4D).

**Pharmacological reduction of IGF-1R in mice receiving high-fat diet leads to reduced hepatic lipid deposition and enhanced insulin mediated Akt phosphorylation.**

Finally, we quantified hepatosteatosis in cixutumumab treated mice in the setting of HFD induced positive energy balance. We found that liver mass (Figure 5A), hepatic triglyceride and cholesterol content were similar in cixutumumab treated mice compared to isotype control treated mice (Figure 5B, 5C). Lipid deposition quantified using haematoxylin and eosin (Figure 5D) and Oil-Red O staining (Figure 5E) was reduced in cixutumumab treated mice compared to isotype control treated mice, as was hepatic lipid droplet area (Figure 5F). Expression of many classical markers of fatty liver disease was similar in cixutumumab-treated mice compared to isotype control-treated mice (Figure 5G) whereas cixutumumab-treated mice showed increased expression of the gene encoding *Mttp* (Figure 5G).

We, then examined the effect of cixutumumab on insulin signalling in liver (Supplementary Figure 6) and muscle (Supplementary Figure 7) of HFD fed mice *in vivo*. To do this, we injected cixutumumab or isotype control treated mice with insulin or vehicle and quantified serine 473 phosphorylation of Akt in liver and skeletal muscle. In cixutumumab-treated mice, basal Akt in liver (Figure 6A) and skeletal muscle (Figure 6D) tended to be lower than isotype control treated mice but this did not reach statistical significance. In cixutumumab-treated mice, phosphorylation of Akt (Figure 6B) showed a strong trend towards an increase in liver, while skeletal muscle showed no difference (Figure 6E). Insulin-stimulated serine 473 phosphorylation of Akt in liver was significantly greater in cixutumumab treated mice compared to isotype control treated mice (Figure 6C) with no significant differences in skeletal muscle (Figure 6F). We, then examined the effect of cixutumumab treatment on responses of molecules downstream of Akt. No significant differences were observed in cixutumumab treated mice in the expression of total FOXO (Supplementary Figure 5A), GSK (Supplementary Figure 5D), or in the phosphorylation levels of FOXO (Supplementary Figure 5B) and GSK (Supplementary Figure 5E). We also observed no significant changes in insulin-mediated phosphorylation of FOXO (Supplementary Figure 5C) or GSK in the cixutumumab treated mice compared to vehicle control (Supplementary Figure 5F).

**Discussion**

To examine the effect of short-term disruption of IGF-1 signalling on increased energy balance-related changes in lipid storage and AT function, we employed a pharmacological approach using the IGF-1R specific antibody cixutumumab, which has been used in humans to treat a range of malignancies ^7, 8^. The following key findings are reported: **1)** Reducing IGF-1R using cixutumumab did not alter IR expression. **2)** Mice on a standard chow diet with reduced IGF-1R expression had no difference in fat depot mass, adipocyte size, hepatic mass or fat content compared to control mice. **3)** In the setting of HFD induced obesity, cixutumumab prevented weight gain and led to a lipodystrophic pattern with reduced WAT expansion and increased lipid deposition in BAT. 4) In the liver of HFD fed mice cixutumumab led to reduced microscopic evidence of hepatosteatosis, increased expression of microsomal triglyceride transfer protein and enhanced insulin stimulated serine phosphorylation of Akt. **5)** In HFD fed mice cixutumumab led to reduced levels of genes encoding proteins important in fatty acid metabolism in subcutaneous and visceral WAT.

**IGF-1R deficient mice develop lipodystrophy on a high fat diet.** When placed on a HFD, mice with reduced IGF-1R due to cixutumumab treatment did not gain as much weight, and displayed increased urinary energy and faecal lipid loss compared to isotype control treated littermates. Consistent with this, mice with reduced levels of the IGF-1R on HFD demonstrated abnormal fat distribution with a reduction in subcutaneous and abdominal fat depot size. In the liver, there was no increase in mass and no significant effect on hepatic triglyceride content. There was however, in cixutumumab treated mice microscopic evidence of reduced hepatosteatosis but no gross effects which may have become more evident with more prolonged feeding. Taken together, this suggests that BAT was the principal site for lipid deposition in IGF-1R deficient mice fed a HFD. Increased hepatic expression of microsomal triglyceride transfer protein, which is essential for the secretion of VLDL ApoB-lipoproteins, may provide an explanation for reduced hepatic lipid droplet formation in cixutumumab recipients. Previous studies have shown that liver-specific microsomal triglyceride transfer protein knockout mice receiving HFD exhibit hepatic steatosis due to ineffective VLDL secretion ^9^, and that obese mice on an ob/ob background have increased hepatic triglyceride content resolved by administration of recombinant adenovirus murine microsomal triglyceride transfer protein DNA ^10^. The relationship between IGF-1 and microsomal triglyceride transfer protein warrants future investigation.

We went on to examine expression of genes important in fatty acid metabolism and flux in different AT depots. Interestingly, we found a depot-specific effect of cixutumumab on these genes, dysregulation of which are thought to contribute to the pathogenesis of the metabolic syndrome ^11^. While we found no difference in plasma free fatty acids in mice fed HFD treated with cixutumumab or isotype control, the finding that cixutumumab treated mice had significantly less visceral and subcutaneous WAT suggests that lipolysis as a measure of total fat mass may be altered in the WAT of cixutumumab treated mice. An effect of reducing IGF-1R in adipocytes on changes in WAT lipolysis in the presence of a high fat diet warrants future study.

**Deficiency of IGF-1R leads to whitening of BAT in the presence of nutritional obesity.** In humans, BAT was thought to disappear during post-natal development, but recent studies have shown that adults harbour BAT ^12^. While a range of studies have examined the mechanisms of ‘browning’ of WAT, information around ‘whitening’ of BAT is limited ^13, 14^. BAT mass was reduced but there was no change in morphology in cixutumumab and isotype control treated obese mice. WAT specific IGF-1R knockout mice receiving standard chow diet had no perturbation of insulin sensitivity or glucose tolerance.

Histological analysis of BAT from cixutumumab treated mice showed adipocytes with a morphology intermediate to typical brown and white adipocytes, along with increased lipid deposition. Multiple mechanisms have been proposed to account for transition between white and brown adipocytes ^15^. In the present report systemic IGF-1R deficiency did not change the expression of archetypical BAT marker genes (e.g. *Ucp-1, Vegf*) in BAT, but did increase peroxisome proliferator activated receptor gamma (*Pparγ*) expression in subcutaneous WAT a well-established regulator of adipocyte expansion and plasticity ^16^.

**Models of adipocyte specific perturbation of IGF-1 signalling compared to whole body reduction in IGF-1R expression.** A number of studies have employed tissue-specific approaches to examine the role of the IGF-1R and its ligand in adipocyte biology. Kloting *et. al.* deleted the IGF-1R in adipocytes using the fatty acid binding protein (*aP2*) promoter ^17^. They found no impact on glucose and insulin tolerance, but showed that these mice had increased WAT mass, with no difference in BAT mass. Interpretation of this report is difficult due to off-target IGF-1R deletion in brain tissue. Moreover, they did not examine the effect of adipocyte specific deletion of the IGF-1R in the setting of increased energy balance.

More recently, Boucher *et. al.* performed studies in mice with adipocyte-specific deletion of both IR and IGF-1R (FIGIRKO) ^18^. On a chow diet FIGIRKO mice had reduced epididymal fat pad mass but unlike our study they observed reduced lipid content of BAT. FIGIRKO were protected against age-induced glucose intolerance, and when fed HFD were protected against obesity with reduced WAT and BAT depot sizes. FIGIRKO receiving HFD unlike the present study were prone to hepatosteatosis. In their study, in contrast to our own findings, Boucher *et. al.* showed that deletion of IGF-1R had minimal effect on WAT expansion, whereas mice lacking IR or both IR and IGF-1R in adipocytes displayed a lipodystrophic phenotype with severe diabetes, insulin resistance and ectopic fat distribution in muscle and liver. BAT mass, in contrast, was only decreased when both IR and IGF-1R were deleted, indicative of a more integrated role of these receptors in BAT physiology.

Sakaguchi *et. al.* ^19^ used tamoxifen-inducible models to avoid potential off-target effects of germline deletion of IR or IGF-1R. Unlike our study, they did not demonstrate a significant effect of reduced adipocyte IGF-1R on fat development. A number of possibilities for the discrepancies between our study and those of Boucher and Sakaguchi should be borne in mind. First, when deleting the IGF-1R, one must consider its effect on insulin signalling. We demonstrated that mice with germline haploinsufficiency of IGF-1R have enhanced insulin-mediated glucose lowering with impaired glucose tolerance ^20^. Studies of osteoblasts ^21^, breast cancer cells ^22^, endothelial cells ^20^, and of particular relevance, brown adipocytes ^23^ have shown that genetic reduction of IGF-1R expression enhances insulin sensitivity, indicating that the insulin-generated signal is negatively regulated by an interaction with the IGF-1R. In cells from humans with polymorphisms associated with a reduction in IGF-1R expression, a similar pattern of enhanced insulin sensitivity has been demonstrated ^24^.

Genetic studies targeting specific cells whilst informative do not provide information regarding the effect of acute whole-body reduction in IGF-1R expression and so potentially may miss disturbances in cross talk between different organs and tissues in the presence of the nutritional challenge of a HFD. Our own study is a case in point, with systemic IGF-1R knockdown leading to reduced WAT expansion in the presence of HFD and evidence suggesting mitigation against hepatic lipid droplet formation supporting an important role for IGF-1R in lipid storage in different organs.

To examine the effect of cixutumumab on hepatic insulin signalling *in vivo,* we injected HFD fed mice with insulin and quantified phosphorylation of the critical signalling molecule Akt in liver and in muscle. We found a strong tendency for a reduction in total Akt in cixutumumab treated mice injected with insulin and consistent with a negative effect of the IGF-1R on insulin signalling described above ^20-24^ enhanced insulin induced Akt serine phosphorylation in obese mice. Consistent with the effects on glucose uptake we showed no increase in insulin mediated phosphorylation of Akt in skeletal muscle. Our findings of a strong tendency for a reduced total pool of Akt in liver in insulin treated mice raises important questions. Previous studies have shown that degradation of Akt may be accompanied by an increase in relative levels of phosphorylated Akt ^25-27^. The role of the IGF-1R in regulation of Akt expression warrants future studies. This notwithstanding these findings raise the intriguing possibility that a potential mechanism underpinning (at least in part) the effect of cixutumumab on hepatic steatosis may be enhanced hepatic insulin sensitivity. Support for this possibility comes from mice with hepatocyte specific deletion of the insulin receptor showing that the key consequence of hepatic insulin resistance, is deleterious cellular and molecular changes which exacerbate hepatocyte triglyceride storage ^28^.

**Potential mechanisms underlying cixutumumab-induced alterations in adipose tissue responses to nutritional obesity.** Changes at a whole-body level affecting multiple tissues mean that a single underpinning mechanism for the lipodystrophy and redistribution of lipid storage seen in cixutumumab-induced IGF-1R deletion is unlikely. While recent publications using gene-modified mice imply a minimal role for IGF-1 in the AT response to an increase in energy balance, our dataset suggest that at a whole-body level this may not be the case. Consistent with this argument, we have previously demonstrated that mice with overexpression of IGF-1 binding protein-2, which limits the access of IGF-1 to its target tissues, have significantly reduced WAT expansion in response to both aging and a high-fat diet ^29^.

The hyperplastic growth of WAT requires the formation of new adipocytes *in vivo*. Because mature adipocytes are post-mitotic, new adipocytes arise from the differentiation of precursor cells residing within AT. Elegant studies from the group of Matthew Rodeheffer show that developmental and obesogenic adipogenesis are regulated through distinct molecular pathways ^30^. Rodeheffer and colleagues showed that HFD in mice rapidly and transiently induced proliferation of adipocyte precursor cells specific to the perigonadal WAT depot in male mice, consistent with the patterns of obesogenic WAT growth seen in humans. Rodeheffer showed in multiple models of obesity that activation of adipocyte precursor cells in diet-induced obesity is dependent on the phosphoinositide 3-kinase-AKT pathway. The effect of cixutumumab and the IGF-1R *per se* in this pathway warrants investigation.

Mice treated with cixutumumab on a high fat diet had a reduction in weight gain and adiposity which may be in part explained by reduced lipid absorption. This raises important question of how does cixutumumab impact on intestinal function. Multiple mechanisms may account for this effect on lipid absorption we recently demonstrated that the IGF-1R in the endothelium may act as a nutrient sensor and when overexpressed in the endothelium may change the architecture of the gut microbiota ^31^

Adipocyte size was reduced by cixutumumab treatment in mice fed a high fat diet the underlying mechanisms may simply be reduced lipid absorption. On the other hand an effect on adipose tissue expandability ^32^ cannot be excluded.

**Study limitations.** The present study used a non-genetic approach to avoid the need for tamoxifen to induce knockdown of IGF-1R and potential off-target effects using a genetic approach. However, cixutumumab induced global reduction of IGF-1R and whole body insulin resistance makes it difficult to dissect the specific role of IGF-1R at a tissue level. Moreover, we did not perform more prolonged studies of cixutumumab administration, so the longer-term effects and the potential for WAT regeneration, as reported by Sakaguchi *et. al.* ^19^ cannot be commented on using the present dataset. We examined the effect of cixutumumab in male mice only. Studies have shown that when controlling for diet and other environmental conditions, male mice show significantly greater expansion of total body mass including subcutaneous AT ^33, 34^. Studies in female mice would be of interest. In the present analysis mice fed HFD treated with cixutumumab ingested more food than isotype control treated mice. Pair feeding experiments limiting the amount of calories to cixutumumab treated mice as ingested by isotype control mice would provide further information on the effects of IGF-1R blockade independent of differences in energy intake. In the present study we did not perform detailed metabolic cage studies so a contribution of increased energy expenditure and changes in locomotor activity cannot be excluded.

**Methods and Materials**

**Animals & animal procedures**. C57BL/6J mice (Purchased from Charles River) were maintained in an Animal Holding; room temperatures were kept at 21⁰C, within a range of +/- 2⁰C and humidity controlled environment on a 12 hour light: dark cycle. Male mice were studied in all experiments, with treatment beginning at 6-8 weeks of age, and were conducted in accordance with accepted standards of humane animal care under United Kingdom Home Office Project license No. P144DDO6.

**Pharmacological reduction of IGF-1R.** To acutely reduce IGF-1R expression, we used cixutumumab (A12), a monoclonal antibody that causes internalisation and degradation of IGF-1R ^7, 8^. Cixutumumab and an isotype control (IC) antibody to an irrelevant antigen (anti-KLHλ IgG1) were obtained from ImClone Systems. 3 weeks after commencing a high-fat diet mice were treated every 3 days with 10mg/kg cixutumumab or isotype control antibody by intraperitoneal (IP) injection for 3 weeks as previously described ^35^ and experimental protocol shown in Supplementary Figure 1A.

**High fat diet induced obesity.** To induce obesity, mice were fed HFD (60%) Diet F3282, 5450 kcal/kg; Bio-serv, Frenchtown NJ; for details of diet composition, see https://www.bio-serv.com/product/HFPellets.html ^29, 36, 37^, as shown in the experimental protocol (Supplementary Figure 1A).

**Mouse growth and morphology.** Body and organ mass was determined after 6 weeks of feeding HFD or standard chow diet. Adipose tissue (epididymal fat pads, subcutaneous depots and interscapular brown fat depots) was carefully dissected, blotted and weighed immediately.

***In vivo* examination of glucose homeostasis and insulin signalling.** *In vivo* metabolic testing was performed as previously described ^23, 36, 37^, for glucose tolerance testing, mice were fasted for 12 hours, followed by intraperitoneal (IP) injection of 1mg/kg glucose. For insulin tolerance testing ^23, 36, 37^, mice were fasted for 4 hours, followed by IP injection of 0.75 units/kg insulin (Actrapid; NovoNordisk, Bagsvaerd, Denmark). Whole-blood glucose was determined at 30 minute intervals by tail vein sampling using a portable meter (Accu-chek Aviva; Roche Diagnostics, Burgess Hill, U.K.). Plasma insulin was measured using ultrasensitive mouse ELISA kit (CrystalChem, Downers Grove, IL), as previously described ^37^. To examine *in vivo* insulin signalling, after 3 weeks of cixutumumab treatment, fasted mice were injected IP with insulin (0.75 U/kg) or vehicle (saline). After 15 min, mice were sacrificed and livers rapidly harvested and snap-frozen.

**Free fatty acids, triglycerides, cholesterol and leptin levels.** Plasma free fatty acids and triglyceride concentrations were determined using colorimetric assays ^37^ (Free Fatty Acids Half-Micro test, Roche, Mannheim, Germany, and Abcam, Cambridge, UK, respectively). Leptin levels were measured using commercially available enzyme-linked immunosorbant assays (Merck Millipore, Germany).

**Fat depot and adipocyte size.** Samples of AT from epididymal fat pads, subcutaneous white AT (WAT) and interscapular brown AT (BAT) depots were fixed in 4% paraformaldyhde and embedded in paraffin. Multiple sections (separated by 100µm each) were obtained from each sample and stained with haematoxylin and eosin. Digital images of each section were acquired; cell areas were traced manually for at least 100 cells per field by an investigator blinded to sample identity, using Image J software. Two fields from each of 2 sections from each adipose tissue depot were analysed to derive the mean cell area per animal ^29^.

**Liver triglyceride content.** Liver triglycerides were determined in 10% liver homogenates prepared in buffer containing 250mM sucrose, 1mM EDTA, 10mM Tris-HCl pH 7.5, using a commercial kit (GPO-PAP, Biolabo, SA Maizy, France). In brief, triglycerides were hydrolysed to glycerol and free fatty acids by lipoprotein lipase (LPL). Further conversion and oxidation by glycerol kinase (GK) and glycerol-3-phosphate (GPO), respectively allowed measurement of triglyceride content at 485nm absorbance.

**Liver histopathology.** Liver samples were fixed in 4% paraformaldehyde, embedded in paraffin, sectioned and the slides rehydrated before hematoxylin and eosin staining. Oil Red O staining was performed on frozen samples. Qualitative and quantitative analysis was performed by two senior consultant histopathologists (JW, RB) blinded to treatments. Lipid droplets were quantified per high-power field ^38^.

**Quantification of receptor and Akt, GSK and FOXO expression.** IGF-1R, IR, phospho-Akt, Akt, phosphor-GSK, GSK, phospho-FOXO and FOXO protein expressions were quantified in tissues by Western blotting [13,31]. Tissues were lysed in extraction buffer containing 50mM HEPES, 120mM NaCl, 1mM MgCl_2_, 1mM CaCl_2_, 10mM NaP_2_O_7_, 20mM NaF,1mM EDTA, 10% glycerol, 1% NP40, 2mM sodium orthovanadate, 0.5μg/ml leupeptin, 0.2mM PMSF, and 0.5μg/ml aprotinin, before protein measurements were carried out by BCA assay (Pierce). Equal amounts of protein were resolved on SDS polyacrylamide gels (Invitrogen) and transferred to polyvinylidine difluoride membranes. Blots were incubated with appropriate primary antibodies (IR-beta (C-19) and IGF-1R-beta (C-20) and β-actin (C-4) from Santa Cruz Biotechnologies); Akt, Ser^473^ pAkt (XP), Phospho-GSK-3α/β (Ser^21/9^:D17D2), GSK-3α (D80E6), Phospho-FOXO (PA5-110122) from ThermoFisher and FOXO (NBP3-05667) from Novus Biologicals), and their corresponding peroxidise-conjugated secondary antibodies. Immunoblots were developed using enhanced chemiluminescence (Millipore) kits. Immunoblots were scanned on a SynGene Imager and bands were quantified using NIH Image J software. Protein expression was normalised to β-actin levels.

**Gene expression.** Adipose tissue RNA was isolated by homogenising with TRI-reagent as previously described ^37^, converted to cDNA using a High Capacity Conversion Kit (Applied Biosystems, Foster City, USA) and assayed using a Lightcycler 480 SYBR Green MasterMix (Roche, Basel, Switzerland). mRNA levels of *Ucp-1, Vegf-1*, *Igf-1r*, *Ir, Lep,* *Cidea, FAS, SREPBP1, ACC1*, *Hmgr,* and *Ppar*γ were quantified and normalised to the housekeeping gene *18S*. Liver RNA was isolated using a Monarch Total RNA Miniprep kit, converted to cDNA with Luna Cell Ready One-Step RT-qPCR (both New England Biolabs, MA, US) and assayed using an iTaq Universal SYBR Green Supermix (BioRad, CA, US). Levels of *Mttp, Hmgr, Srebp1c, Ppara, Cpt1a, Acc1, Fas, Chrebp, Cypp7A1, Hif1a, Tnfa* were quantified and normalised to *rps29.* In both cases real-time quantitative PCR was performed by Roche LIGHTCYCLER 480 in a 384 well format ^20^. Primer sequences are shown in Table 2.

**Metabolic assessment.** To examine food and water intake, urine output and faecal mass, mice were housed in individual cages (Techniplast no. 3700M071, Scanbur, Koge, Denmark) and acclimatised for 2 days before commencing urine and faeces collection and monitoring food intake for 5 consecutive days. Body temperature was measured using a rectal thermometer, as previously reported ^29^.

**Energy lost in urine.** Urine samples were deproteinised using a TCA kit then assayed for glucose content with a commercial kit (both Abcam, Cambridge, UK). Energy loss in urine was calculated using the following calculations: Energy lost in urine kJ/day = (glucose in urine [mMol/l]/1,000) × molecular weight glucose × (water intake [ml/day]/1,000) × E density_carb_; E density_carb_ = energy density related to oxidations within the body for carbohydrates as glucose = 15.76 kJ/g ^16^.

**Faecal lipid loss.** Faecal samples (100mg) were ground and lipids extracted with saline and chloroform:methanol (2:1). After centrifugation, the lower lipid phase was transferred to pre-weighed tubes and after overnight evaporation, the weight differential was used to calculate the lipid mass per 1000mg of faeces and average faecal lipid excretion per day.

**Statistics.** Results are expressed as mean (SEM). Comparisons within groups were made using paired Students t tests and between groups using unpaired Students t tests or area under curve ^39^, as appropriate, *n* denotes number of mice per group per experiment.

**Acknowledgements.**

Technical support provided by Jessica Smith.

**Funding**

This work was supported by the British Heart Foundation. MTK is British Heart Foundation Professor of Cardiovascular and Diabetes research; SBW was supported by a European Research Council starter award 310747. RMC was supported by British Heart Foundation Intermediate Research Fellowship FS/12/80/29821.

**Author contributions**.

HI, HV, KIB, NJH, NYY, SG, KJS, NTW, LL, HK and BR contributed to the acquisition, analysis and interpretation of data. JW and RG provided additional expert analysis. HI, HV, RMC, PS, SBW. MTK contributed to the conception, design, draft and revision of the work.

**Conflict of Interest Statement**

The authors declare no competing interests.

**Data Availability Statement**

Datasets would be available upon formal request from the corresponding author.

References

1. Spiegelman BM, Flier JS. Obesity and the regulation of energy balance. Cell 2001; 104:531-43.

2. Kahn BB, Flier JS. Obesity and insulin resistance. J Clin Invest 2000; 106:473-81.

3. Imrie H, Abbas A, Kearney M. Insulin resistance, lipotoxicity and endothelial dysfunction. Biochim Biophys Acta 2010; 1801:320-6.

4. Narasimhan SD, Yen K, Tissenbaum HA. Converging pathways in lifespan regulation. Curr Biol 2009; 19:R657-66.

5. Tan KT, Luo SC, Ho WZ, Lee YH. Insulin/IGF-1 receptor signaling enhances biosynthetic activity and fat mobilization in the initial phase of starvation in adult male C. elegans. Cell Metab 2011; 14:390-402.

6. Boucher J, Charalambous M, Zarse K, Mori MA, Kleinridders A, Ristow M, et al. Insulin and insulin-like growth factor 1 receptors are required for normal expression of imprinted genes. Proc Natl Acad Sci U S A 2014; 111:14512-7.

7. Rajan A, Carter CA, Berman A, Cao L, Kelly RJ, Thomas A, et al. Cixutumumab for patients with recurrent or refractory advanced thymic epithelial tumours: a multicentre, open-label, phase 2 trial. Lancet Oncol 2014; 15:191-200.

8. Rowinsky EK, Schwartz JD, Zojwalla N, Youssoufian H, Fox F, Pultar P, et al. Blockade of insulin-like growth factor type-1 receptor with cixutumumab (IMC-A12): a novel approach to treatment for multiple cancers. Curr Drug Targets 2011; 12:2016-33.

9. Newberry EP, Xie Y, Kennedy SM, Graham MJ, Crooke RM, Jiang H, et al. Prevention of hepatic fibrosis with liver microsomal triglyceride transfer protein deletion in liver fatty acid binding protein null mice. Hepatology 2017; 65:836-52.

10. Chen Z, Newberry EP, Norris JY, Xie Y, Luo J, Kennedy SM, et al. ApoB100 is required for increased VLDL-triglyceride secretion by microsomal triglyceride transfer protein in ob/ob mice. J Lipid Res 2008; 49:2013-22.

11. Samuel VT, Shulman GI. The pathogenesis of insulin resistance: integrating signaling pathways and substrate flux. J Clin Invest 2016; 126:12-22.

12. Cypess AM, Lehman S, Williams G, Tal I, Rodman D, Goldfine AB, et al. Identification and importance of brown adipose tissue in adult humans. N Engl J Med 2009; 360:1509-17.

13. Mori MA, Thomou T, Boucher J, Lee KY, Lallukka S, Kim JK, et al. Altered miRNA processing disrupts brown/white adipocyte determination and associates with lipodystrophy. J Clin Invest 2014; 124:3339-51.

14. Shimizu I, Aprahamian T, Kikuchi R, Shimizu A, Papanicolaou KN, MacLauchlan S, et al. Vascular rarefaction mediates whitening of brown fat in obesity. J Clin Invest 2014; 124:2099-112.

15. Jeremic N, Chaturvedi P, Tyagi SC. Browning of White Fat: Novel Insight Into Factors, Mechanisms, and Therapeutics. J Cell Physiol 2017; 232:61-8.

16. Medina-Gomez G, Gray SL, Yetukuri L, Shimomura K, Virtue S, Campbell M, et al. PPAR gamma 2 prevents lipotoxicity by controlling adipose tissue expandability and peripheral lipid metabolism. PLoS Genet 2007; 3:e64.

17. Kloting N, Koch L, Wunderlich T, Kern M, Ruschke K, Krone W, et al. Autocrine IGF-1 action in adipocytes controls systemic IGF-1 concentrations and growth. Diabetes 2008; 57:2074-82.

18. Boucher J, Mori MA, Lee KY, Smyth G, Liew CW, Macotela Y, et al. Impaired thermogenesis and adipose tissue development in mice with fat-specific disruption of insulin and IGF-1 signalling. Nat Commun 2012; 3:902.

19. Sakaguchi M, Fujisaka S, Cai W, Winnay JN, Konishi M, O'Neill BT, et al. Adipocyte Dynamics and Reversible Metabolic Syndrome in Mice with an Inducible Adipocyte-Specific Deletion of the Insulin Receptor. Cell Metab 2017; 25:448-62.

20. Abbas A, Imrie H, Viswambharan H, Sukumar P, Rajwani A, Cubbon RM, et al. The insulin-like growth factor-1 receptor is a negative regulator of nitric oxide bioavailability and insulin sensitivity in the endothelium. Diabetes 2011; 60:2169-78.

21. Fulzele K, DiGirolamo DJ, Liu Z, Xu J, Messina JL, Clemens TL. Disruption of the insulin-like growth factor type 1 receptor in osteoblasts enhances insulin signaling and action. J Biol Chem 2007; 282:25649-58.

22. Zhang H, Pelzer AM, Kiang DT, Yee D. Down-regulation of type I insulin-like growth factor receptor increases sensitivity of breast cancer cells to insulin. Cancer Res 2007; 67:391-7.

23. Entingh-Pearsall A, Kahn CR. Differential roles of the insulin and insulin-like growth factor-I (IGF-I) receptors in response to insulin and IGF-I. J Biol Chem 2004; 279:38016-24.

24. Raile K, Klammt J, Schneider A, Keller A, Laue S, Smith R, et al. Clinical and functional characteristics of the human Arg59Ter insulin-like growth factor i receptor (IGF1R) mutation: implications for a gene dosage effect of the human IGF1R. J Clin Endocrinol Metab 2006; 91:2264-71.

25. Risso G, Blaustein M, Pozzi B, Mammi P, Srebrow A. Akt/PKB: one kinase, many modifications. Biochem J 2015; 468:203-14.

26. Wei Y, Zhou J, Yu H, Jin X. AKT phosphorylation sites of Ser473 and Thr308 regulate AKT degradation. Biosci Biotechnol Biochem 2019; 83:429-35.

27. Chan CH, Jo U, Kohrman A, Rezaeian AH, Chou PC, Logothetis C, et al. Posttranslational regulation of Akt in human cancer. Cell Biosci 2014; 4:59.

28. Michael MD, Kulkarni RN, Postic C, Previs SF, Shulman GI, Magnuson MA, et al. Loss of insulin signaling in hepatocytes leads to severe insulin resistance and progressive hepatic dysfunction. Mol Cell 2000; 6:87-97.

29. Wheatcroft SB, Kearney MT, Shah AM, Ezzat VA, Miell JR, Modo M, et al. IGF-binding protein-2 protects against the development of obesity and insulin resistance. Diabetes 2007; 56:285-94.

30. Jeffery E, Church CD, Holtrup B, Colman L, Rodeheffer MS. Rapid depot-specific activation of adipocyte precursor cells at the onset of obesity. Nat Cell Biol 2015; 17:376-85.

31. Haywood NJ, Luk C, Bridge KI, Drozd M, Makava N, Skromna A, et al. Endothelial IGF-1 receptor mediates crosstalk with the gut wall to regulate microbiota in obesity. EMBO Rep 2021; 22:e50767.

32. Haczeyni F, Bell-Anderson KS, Farrell GC. Causes and mechanisms of adipocyte enlargement and adipose expansion. Obes Rev 2018; 19:406-20.

33. Stubbins RE, Holcomb VB, Hong J, Nunez NP. Estrogen modulates abdominal adiposity and protects female mice from obesity and impaired glucose tolerance. Eur J Nutr 2012; 51:861-70.

34. Griffin C, Lanzetta N, Eter L, Singer K. Sexually dimorphic myeloid inflammatory and metabolic responses to diet-induced obesity. Am J Physiol Regul Integr Comp Physiol 2016; 311:R211-6.

35. Ulanet DB, Ludwig DL, Kahn CR, Hanahan D. Insulin receptor functionally enhances multistage tumor progression and conveys intrinsic resistance to IGF-1R targeted therapy. Proc Natl Acad Sci U S A 2010; 107:10791-8.

36. Imrie H, Abbas A, Viswambharan H, Rajwani A, Cubbon RM, Gage M, et al. Vascular insulin-like growth factor-I resistance and diet-induced obesity. Endocrinology 2009; 150:4575-82.

37. Noronha BT, Li JM, Wheatcroft SB, Shah AM, Kearney MT. Inducible nitric oxide synthase has divergent effects on vascular and metabolic function in obesity. Diabetes 2005; 54:1082-9.

38. Levene AP, Kudo H, Armstrong MJ, Thursz MR, Gedroyc WM, Anstee QM, et al. Quantifying hepatic steatosis - more than meets the eye. Histopathology 2012; 60:971-81.

39. Watt NT, Gage MC, Patel PA, Viswambharan H, Sukumar P, Galloway S, et al. Endothelial SHIP2 Suppresses Nox2 NADPH Oxidase-Dependent Vascular Oxidative Stress, Endothelial Dysfunction, and Systemic Insulin Resistance. Diabetes 2017; 66:2808-21.

**Table Legends**

**Table 1.** Circulating levels of IGF-1, insulin and leptin in cixutumumab treated chow-fat fed mice in fed state. (A12 denotes cixutumumab, IC denotes isotype control, data expressed as mean (SEM)* denotes P<0.05,).

**Table 2. qPCR primers used in the study.**

**Figure legends**

**Figure 1.** Examination of the effect of cixutumumab (A12) a specific antibody leading to internalisation and degradation of the insulin like growth factor-1 receptor (IGF-1R) in chow fed mice. Male C57BL/6J mice were fed standard chow for 6-weeks and received 10mg/Kg cixutumumab or isotype control (IC) every 3 days by intraperitoneal injection for 3 weeks, 21 days after commencing diet. **A)** No difference in body weight in cixutumumab treated mice compared to isotype control treated mice (*n*=5). **B)** No difference in organ weight in cixutumumab treated mice compared to isotype control treated mice (*n*=26). **C)** No difference in epididymal fat pad (EFP) weight in cixutumumab treated mice compared to isotype control (*n*=26). **D)** No change in white adipocyte size in EFP from cixutumumab treated mice compared to isotype control (n=12). **E)** No change in brown adipose tissue (BAT) weight in cixutumumab treated mice compared to isotype control treated mice (*n*=18). **F)** No difference in glucose tolerance tests in cixutumumab treated mice compared to isotype control treated mice (*n*=8). **G)** Insulin tolerance tests in cixutumumab treated mice demonstrated a blunted decline in blood glucose in response to insulin compared to isotype control-treated mice (*n*=8). **H)** No difference in liver weight in cixutumumab treated mice compared to isotype control treated mice (*n*=26). **I)** No difference in hepatic triglyceride content in cixutumumab treated mice compared to isotype control treated mice (*n*=5). **J)** No difference in hepatic cholesterol content in cixutumumab treated mice compared to isotype control treated mice (*n*=5). Data expressed as mean (SEM),* denotes P<0.05, *n* denotes number of mice per group, comparisons made using unpaired students t test or area under curve (AUC) where indicated.

**Figure 2.** Examination of the effect of cixutumumab (A12) a specific antibody leading to internalisation and degradation of the insulin like growth factor-1 receptor (IGF-1R) in high fat fed mice. Male C57BL/6J mice were fed a high fat diet for 6-weeks and received 10mg/Kg cixutumumab or isotype control (IC) every 3 days by intraperitoneal injection for 3 weeks, 21 days after commencing diet: **A)** Weight gain in cixutumumab treated mice trended to be reduced compared to isotype control treated mice (Chow; n=5 and High fat; *n*=26). **B)** No difference in organ weight in cixutumumab treated mice compared to isotype control treated mice (*n*=26). **C)** Reduced epididymal fat pad (EFP) weight in cixutumumab treated mice compared to isotype control treated mice (*n*=26). **D)** No difference in brown adipose tissue (BAT) weight in cixutumumab treated mice compared to isotype control treated mice (*n*=18). **E)** Reduced white adipocyte size in EFP from cixutumumab treated mice compared to isotype control mice (*n*=12). **F)** Reduced white adipocyte size in subcutaneous fat depots of cixutumumab treated mice compared to isotype control treated mice (*n*=4). **G)** Increased in white lipid area of BAT in cixutumumab treated mice compared to isotype control treated mice (*n*=7)**. H)** No difference in glucose tolerance tests in cixutumumab treated mice compared to isotype control treated mice (*n*=8). **I)** In insulin tolerance tests, cixutumumab treated mice demonstrated a trend towards a blunted decline in blood glucose in response to insulin compared to isotype control treated mice (*n*=8). Data expressed as mean (SEM),* denotes P<0.05, *n* denotes number of mice per group, comparisons made using unpaired students t test or area under curve (AUC) where indicated.

**Figure 3.** Examination of the metabolic effects of cixutumumab (A12) a specific antibody leading to internalisation and degradation of the insulin like growth factor-1 receptor (IGF-1R) compared to isotype control in high-fat diet fed mice. Male C57BL/6J mice were fed a high fat diet for 6-weeks and received 10mg/Kg cixutumumab or isotype control (IC) every 3 days by intraperitoneal injection for 3 weeks, 21 days after commencing diet: **A)** Increased food intake in cixutumumab treated mice compared to isotype control treated mice (*n*=12). **B)** Similar water intake in A12 treated mice compared to isotype control treated mice (*n*=12). **C)** Tendency for increased urine output in cixutumumab treated mice compared to isotype control treated mice (*n*=12). **D)** Increased faecal output in cixutumumab treated mice compared to isotype control treated mice (*n*=12). **E)** Increased urinary energy loss per day in cixutumumab treated mice compared to isotype control treated mice (*n*=12). **F)** Increased faecal lipid loss per day in cixutumumab treated mice compared to isotype control treated mice (*n*=12). **G)** Gene expression in epididymal fat pad (EFP) of cixutumumab treated mice compared to isotype control treated mice (*n*=9-13). **H)** Gene expression in brown adipose tissue (BAT) from cixutumumab treated mice compared to isotype control treated mice (*n*=15-20). **I)** Gene expression in BAT from cixutumumab treated mice compared to isotype control treated mice (*n*=11-13). **J)** *Pparg* gene expression was increased in subcutaneous adipose tissue (ScAT) of cixutumumab treated mice compared to isotype control treated mice (*n*=11-13). Data expressed as mean (SEM),* denotes P<0.05, *n* denotes number of mice per group, comparisons made using unpaired students t test or area under curve (AUC) where indicated.

**Figure 4.** Examination of the effects of cixutumumab (A12) a specific antibody leading to internalisation and degradation of the insulin like growth factor-1 receptor (IGF-1R) on expression of mRNAs encoding genes important in fatty acid and cholesterol metabolism. Male C57BL/6J mice were fed a high fat diet for 6-weeks and received 10mg/Kg A12 or isotype control (IC) every 3 days by intraperitoneal injection for 3 weeks, 21 days after commencing diet: **A)** No difference in expression of *pparg* in epididymal white adipose tissue of cixutumumab treated mice compared to isotype control treated mice (*n*=9-12). **B)** No difference in mRNAs of *Mttp, Hmgr, Fas, Srebp1c or Acc1* in brown adipose tissue of cixutumumab treated mice compared to isotype control treated mice. **C)** Reduced mRNAs of *Mttp, Hmgr and Acc1 in* subcutaneous white adipose tissue of cixutumumab treated mice compared to isotype control treated mice (*n*=7-10). **D)** Reduced mRNA of *Mttp* in epididymal WAT of cixutumumab treated mice compared to isotype control treated mice (*n*=5-7). Data expressed as mean (SEM),* denotes P<0.05; **P< 0.005; ^#^P<0.05. *n* denotes number of mice per group, comparisons made using unpaired students t test.

**Figure 5.** Examination of the effects of cixutumumab (A12) a specific antibody leading to internalisation and degradation of the insulin like growth factor-1 receptor (IGF-1R) compared to isotype control on hepatosteatosis in high fat fed mice. Male C57BL/6J mice were fed a high fat diet for 6-weeks and received 10mg/Kg cixutumumab or isotype control (IC) every 3 days by intraperitoneal injection for 3 weeks, 21 days after commencing diet: **A)** No difference in liver weight in cixutumumab treated mice compared to isotype control treated mice (*n*=26). **B)** No difference in hepatic triglyceride in cixutumumab treated mice compared to isotype control treated mice (*n*=5). **C)** Tendency for increased hepatic cholesterol content in cixutumumab treated mice compared to isotype control treated mice (*n*=5). **D)** Decreased number of lipid vacuoles in H&E stained sections of liver from cixutumumab treated mice compared to isotype control treated mice (n=6). **E)** Reduced mean steatosis area in liver quantified using Oil-Red O staining in cixutumumab treated mice compared to isotype control treated mice (*n*=4). **F)** Reduced mean vacuole area in cixutumumab treated mice compared to isotype control treated mice (*n*=6)**.** **G)** Expression levels of fatty liver markers in liver tissue of cixutumumab treated mice compared to isotype control treated mice (*n*=5-9) showing a significant increase in *Mttp* mRNA. Data expressed as mean (SEM),* denotes P<0.05, *n* denotes number of mice per group, comparisons made using unpaired students t test.

**Figure 6.** Examination of the effects of cixutumumab (A12) a specific antibody leading to internalisation and degradation of the insulin like growth factor-1 receptor (IGF-1R) on insulin induced serine-473 phosphorylation of Akt in liver and muscle in fasted mice. Male C57BL/6J mice were fed a high fat diet for 6-weeks and received 10mg/kg cixutumumab or isotype control (IC) every 3 days by intraperitoneal injection for 3 weeks, 21 days after commencing diet: Isotype Control (IsoC) and cixutumumab (A12)-treated high fat-fed mice were injected with Insulin (Ins; (0.75 unit/kg: Actrapid, *n*=5) or vehicle control (C; *n*=5). **A)** No significant difference in total Akt expression in liver between cixutumumab (A12) and isotype control (IsoC) treated mice fed high fat diet (*n*=5 each group) injected with vehicle (*n*=5). **B)** There was a strong statistical trend towards increased phosphorylation of Akt (Akt-Ser473) in liver between cixutumumab (A12) and isotype control (IsoC) treated mice on high fat diet injected with vehicle control (*n*=5). **C)** Insulin-stimulated (Ins) Serine-473 phosphorylated Akt (Akt-Ser473) was significantly greater in liver of cixutumumab treated (A12) mice compared to isotype control (Iso C) treated mice fed high fat diet (*n*=5). **D)** No significant difference in total Akt expression in muscle between cixutumumab (A12) and isotype control (IsoC) treated mice fed high fat diet (*n*=5) injected with vehicle. **E)** No significant difference was observed in the phosphorylation levels of Akt (Akt-Ser473) in muscle between cixutumumab (A12) and isotype control (IsoC) treated mice on high fat diet injected with vehicle control (*n*=5). **F)** No statistical difference was seen in the insulin-stimulated (Ins) Akt phosphorylation in muscle of cixutumumab treated (A12) mice compared to isotype control (Iso C) treated mice fed high fat diet (*n*=5). Representative blot shown in the left-most panels of Akt. Akt-Ser473/Relative Akt expression was calculated as Akt-Ser473 / (Akt/Actin). Data expressed as mean (SEM),* denotes P<0.05, *n* denotes number of mice per group, comparisons made using unpaired students t test, using Mann Whitney test.
