# Supplementary figures and images for: Cixutumumab reveals a critical role for IGF-1 in adipose and hepatic tissue remodelling during the development of diet-induced obesity
Source: Adipocyte. 2022 Jun 23;11(1):366–78. doi: 10.1080/21623945.2022.2089394 (PMC9235901; doi:10.1080/21623945.2022.2089394)

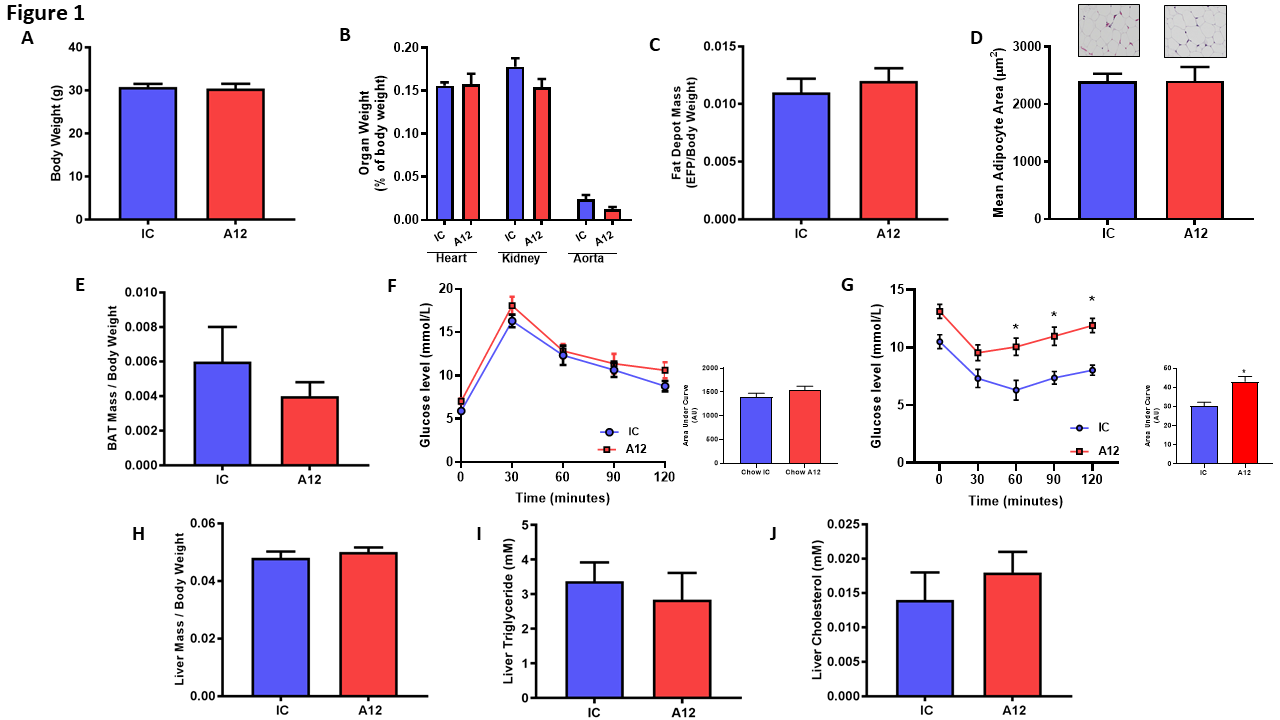

Supplement: Supplemental Material [file KADI_A_2089394_SM2687.zip › Slide1.TIF]

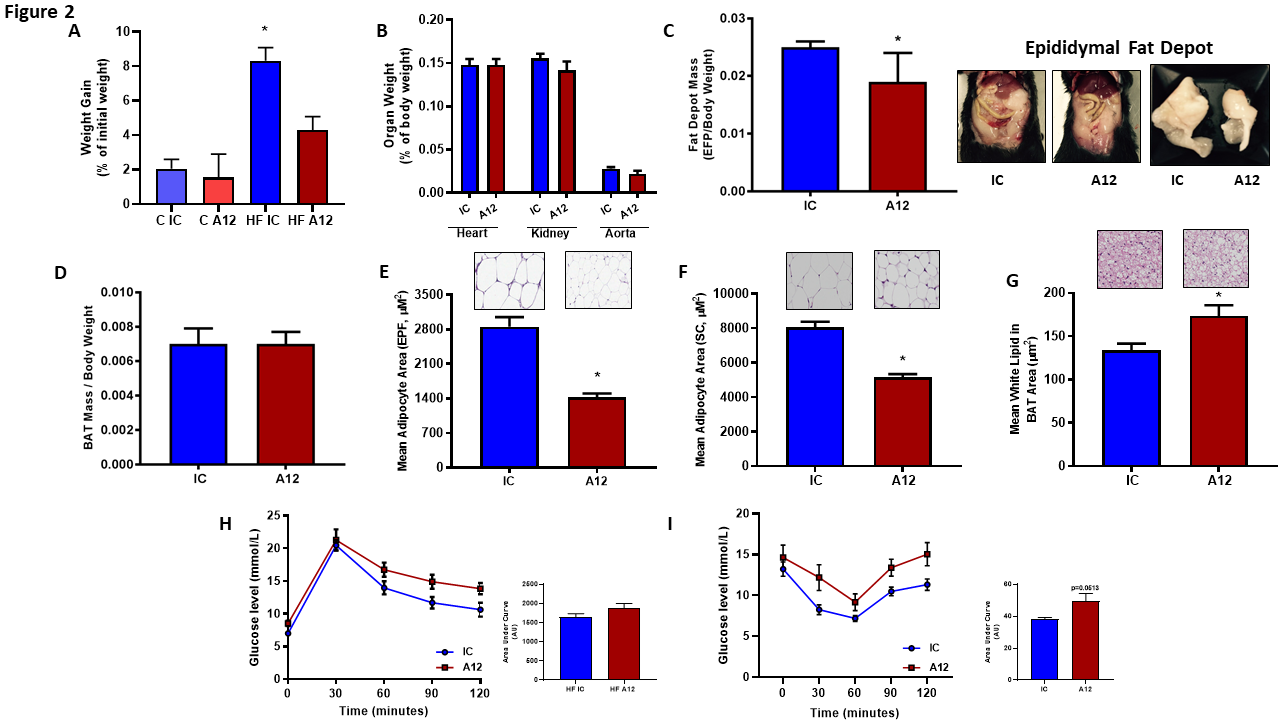

Supplement: Supplemental Material [file KADI_A_2089394_SM2687.zip › Slide2.TIF]

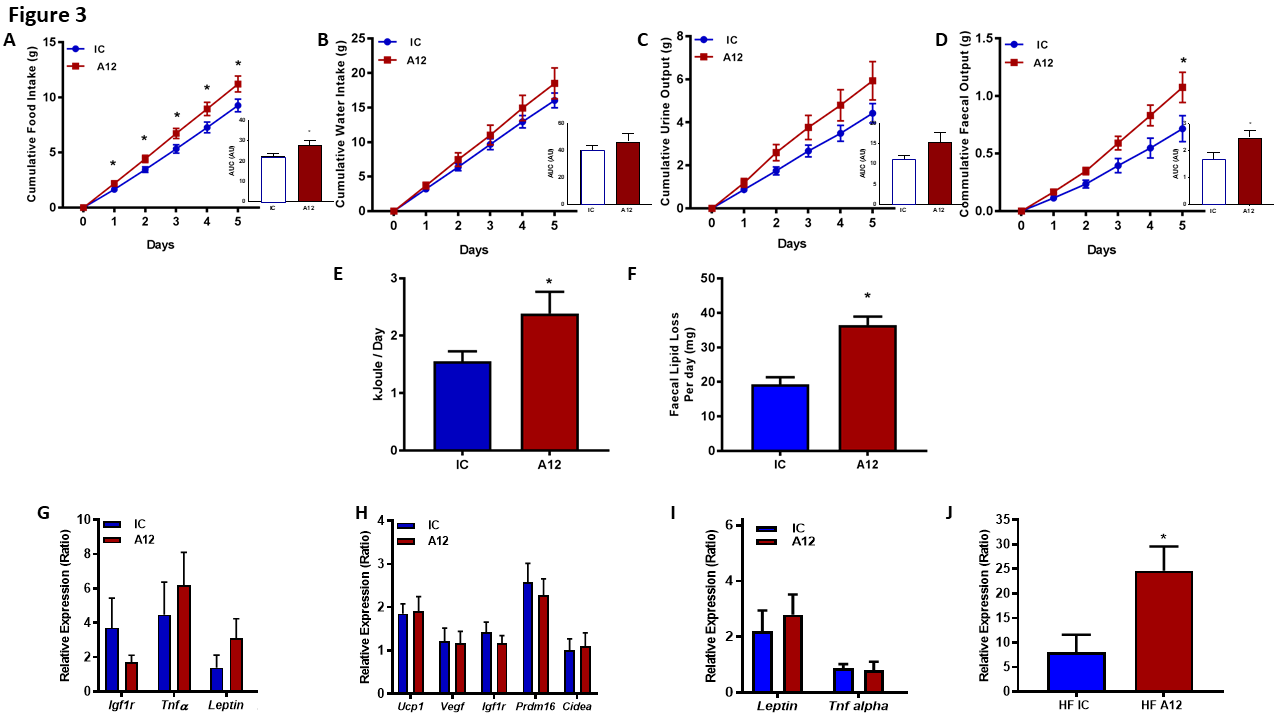

Supplement: Supplemental Material [file KADI_A_2089394_SM2687.zip › Slide3.TIF]

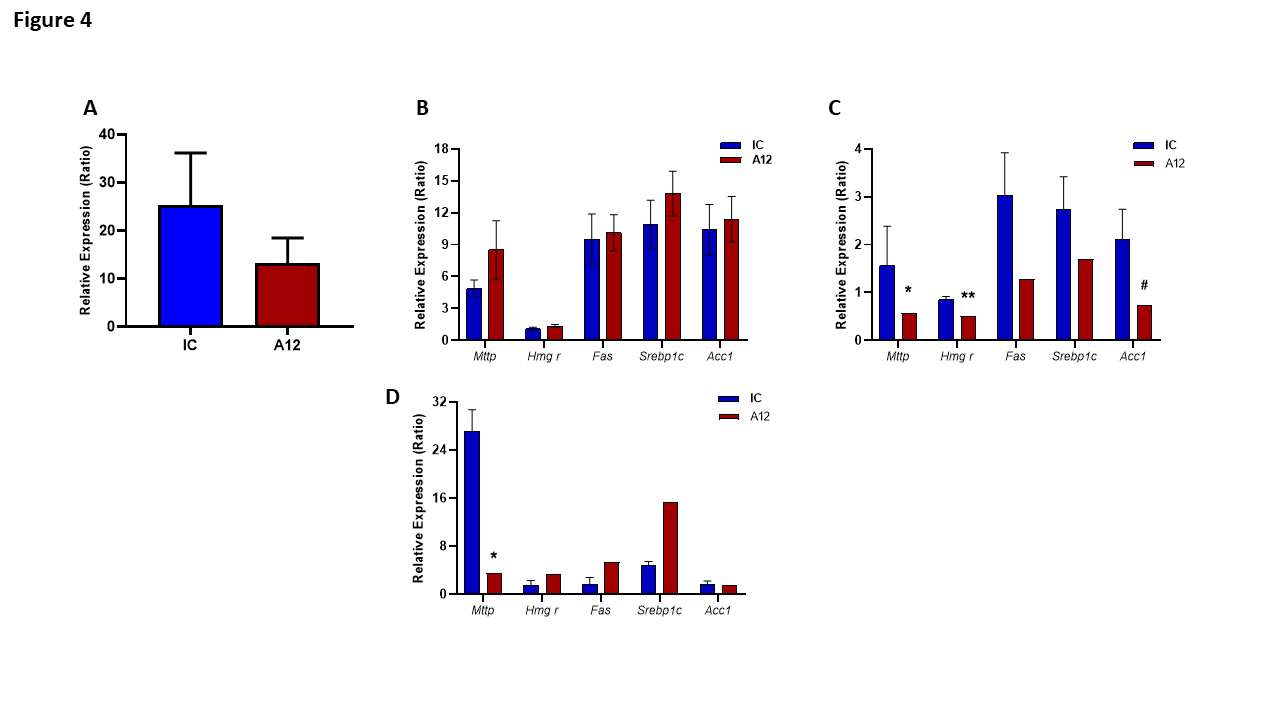

Supplement: Supplemental Material [file KADI_A_2089394_SM2687.zip › Slide4.TIF]

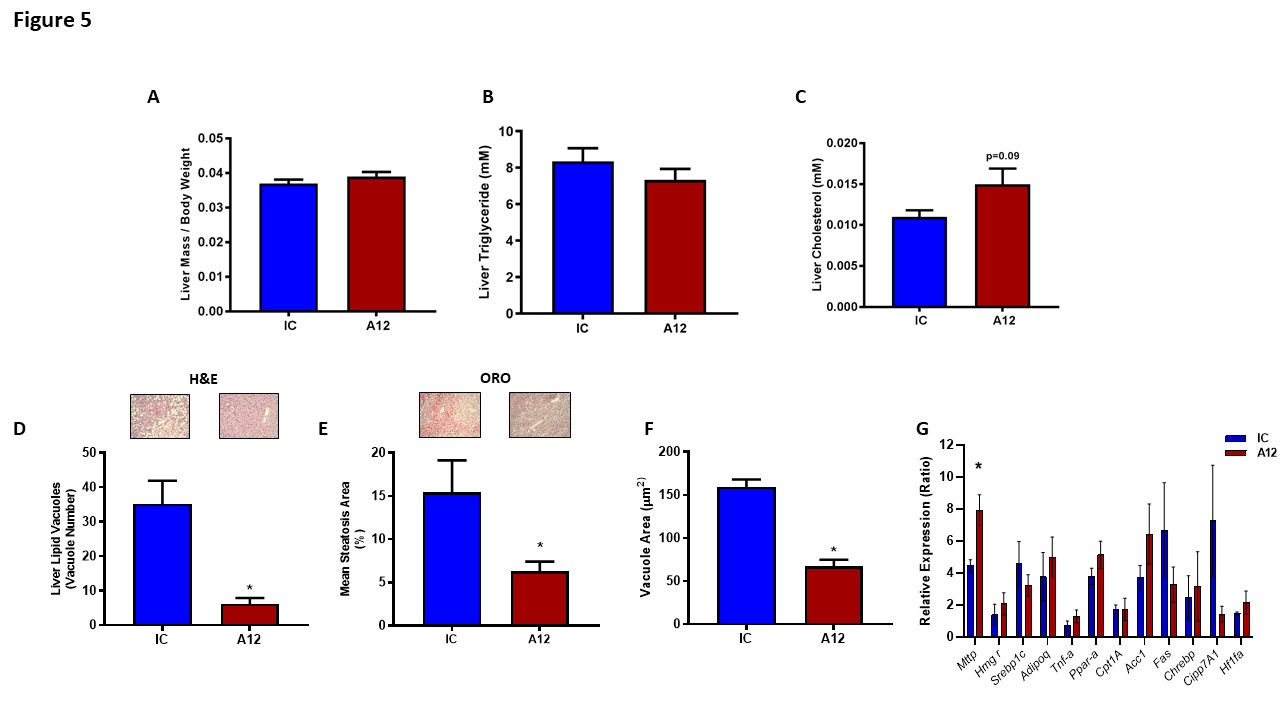

Supplement: Supplemental Material [file KADI_A_2089394_SM2687.zip › Slide5.TIF]

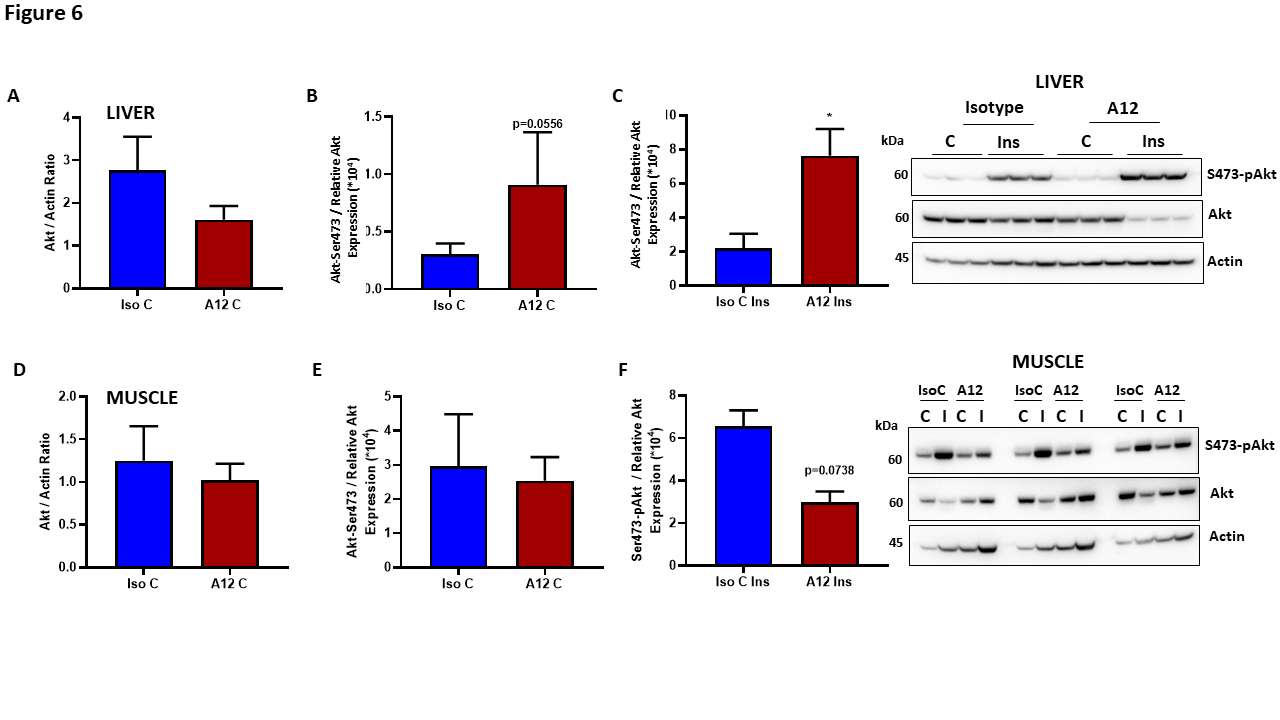

Supplement: Supplemental Material [file KADI_A_2089394_SM2687.zip › Slide6.TIF]
