# Supplementary material for: Cixutumumab reveals a critical role for IGF-1 in adipose and hepatic tissue remodelling during the development of diet-induced obesity
Source: Adipocyte. 2022 Jun 23;11(1):366–78. doi: 10.1080/21623945.2022.2089394 (PMC9235901; doi:10.1080/21623945.2022.2089394)
Supplement: Supplemental Material [file KADI_A_2089394_SM2687.zip › 224363545 Adipocyte Supplementary R1.doc]

**Supplementary Figure 1:** Examination of the effect of cixutumumab (A12) a specific antibody leading to internalisation and degradation of the insulin like growth factor-1 receptor (IGF-1R) in standard chow and

high fat fed male C57BL/6J mice: **A)** Treatment schedule. Mice were fed standard chow or high fat diet for 6-weeks and received 10mg/Kg cixutumumab or isotype control (IC) every 3 days by intraperitoneal injection for

a 3-week period from 3 weeks onwards. **B)** Cixutumumab treated chow fat fed mice had a reduction in IGF-1R expression in liver, fat and muscle (*n*=7-9). **C)** No effect of cixutumumab on insulin receptor (IR) expression in liver, epididymal fat, and muscle in chow-fed mice (*n*=7-9). **D)** Cixutumumab treated high-fat fed mice had a reduction of IGF-1R expression in liver, epididymal fat and muscle (*n*=7-9). **E)** No effect of cixutumumab on IR expression in liver, epididymal fat or muscle in high-fat fed mice (*n*=7-9). Data expressed as mean (SEM)* denotes P<0.05, *n* denotes number of mice per group, comparisons made using unpaired students t test).

**A**

| **High-fat/chow** | GTT/ITT |  |  |  | **A12** |  |  |  | GTT/ITT | Organharvest |
| --- | --- | --- | --- | --- | --- | --- | --- | --- | --- | --- |
|  |  |  |  |  |  |  |  |  |  |  |
|  |  | 10mg/kg **A12** or **Isotype control (IC)** | | | | | | |  |  |
| Day 0 |  |  |  |  |  |  |  |  |  |  |
|  |  |  |  |  |  |  |  |  |  |  |
|  | 21 | 23 | | 27 | 30 | 34 | 37 |  | 41 |  |

**C**	**D**

**5**

| **/Actin** |  | **4** |
| --- | --- | --- |
| **Expression** | **(Ratio)** |  |
|  |  | **3** |
|  |  |  |
|  |  | **2** |
| **1R** |  | **1** |
| **IGF-** |  |  |
|  |  |  |
|  |  | **0** |

| **B** |  | **2.5** |  |  |  | **IC** |  |  | **Liver** |  |  |
| --- | --- | --- | --- | --- | --- | --- | --- | --- | --- | --- | --- |
|  |  |  |  |  |  |  |  |  |  |  |  |
| **/Actin** |  | **2.0** |  |  |  | **A12** | **C** | **C** | **HF** |  | **HF** |
|  |  |  |  |  |  |  |  |  |  |  |  |
|  |  |  |  |  |  |  |  |  |  |  |  |
| **expression** | **(Ratio)** | **1.5** |  |  |  |  | **A12** | **IC** | **A12** |  | **IC** |
|  |  |  |  |  |  |  |  |  |  |  | **IGF1-Rb** |
|  |  |  |  |  |  |  |  |  |  |  |  |
|  |  |  |  |  |  |  |  |  |  |  |  |
|  |  | **1.0** |  |  | ***** |  |  |  |  |  | **IRb** |
| **1R** |  |  |  |  |  | ***** |  |  |  |  |  |
|  |  |  | ***** |  |  |  |  |  |  |  |  |
|  |  | **0.5** |  |  |  |  |  |  |  |  |  |
| **IGF-** |  |  |  |  |  |  |  |  |  |  | **b-actin** |
|  |  |  |  |  |  |  |  |  |  |  |  |
|  |  | **0.0** |  |  |  |  |  |  |  |  |  |
|  |  |  | **Liver** | **Fat** | | **Muscle** |  |  |  |  |  |
|  |  |  |  | **E** |  |  |  | **Fat** | | **Muscle** | |
|  |  |  |  |  | **3** |  |  |  |  |  |  |
|  |  |  | **IC** |  |  |  | **IC** |  |  |  |  |
|  |  |  |  |  |  |  |  |  |  |  |  |
|  |  |  | **A12** |  |  |  | **A12** | **HF** | **HF** | **HF** | **HF** |
|  |  |  |  | **/ Actin** |  |  |  |  |  |  |  |
|  |  |  |  |  |  |  |  |  |  |  |  |
| ***** |  |  |  |  | **2** |  |  | **IC** | **A12** | **IC** | **A12** |
|  |  |  |  | **Expression** | **(Ratio)** |  |  |  |  |  | **IRb** |
|  |  |  |  |  |  |  |  |  |  |  | **IGF-1Rb** |
|  | ***** |  |  |  | **1** |  |  |  |  |  |  |
|  |  |  |  |  |  |  |  |  |  |  |  |
|  |  |  | ***** | **IR** |  |  |  |  |  |  |  |
|  |  |  |  |  |  |  |  |  |  |  | **b actin** |
|  |  |  |  |  | **0** |  |  |  |  |  |  |
|  |  |  |  |  |  |  |  |  |  |  |  |

**Liver**	**Fat**	**Muscle**	**Liver**	**Fat**	**Muscle**

**Supplementary Figure 2:** Examination of the effect of cixutumumab (A12) a specific antibody leading to internalisation and degradation of the insulin like growth factor-1 receptor (IGF-1R) in standard chow and high fat fed male C57BL/6J mice: **A)** Body weight evolution of Chow (C) fed with isotype control (IC) and cixutumumab (A12)-fed vs high fat (HF) fed mice (n=26). **B)** Area Under the curve for Body weight of Chow (C, n=5) fed with isotype control (IC) and cixutumumab (A12)-fed mice vs high fat (HF) fed mice (n=26). **C)** Final body weight of high fat (HF) fed mice with isotype control (IC) and cixutumumab (A12) (n=26). **D)** Fasting glucose levels of Chow-fed mice with isotype control (IC) and cixutumumab (A12) n=8 **E)** Fasting glucose levels of High Fat-fed mice with isotype control (IC) and cixutumumab (A12) n=8. Data expressed as mean (SEM)* denotes P<0.05, *n* denotes number of mice per group, comparisons made using unpaired students t test.

**A**

|  | **35** |
| --- | --- |
| **(g)** | **30** |
| **BodyWeight** |  |
|  | **20** |
|  | **25** |
|  | **15** |
|  | **0** |

**12**	**24**	**36**

**Days**

C **IC**

**C A12 HF IC HF A12**

**48**

**B**	**C**

|  |  | **1500** |  | ***** |
| --- | --- | --- | --- | --- |
|  |  |  |  |  |
| **Curve** |  | **1250** | ***** |  |
|  |  |  |  |  |
|  |  |  |  |  |
| **Under** | **(AU)** | **1000** |  |  |
|  |  |  |  |  |
| **Area** |  | **750** |  |  |
|  |  |  |  |  |
|  |  | **500** |  |  |
|  |  | **C IC** | **C A12** | **HF IC HF A12** |

**D**

|  |  | **15** |
| --- | --- | --- |
| **FastingGlucose** | **(mmol/L)** | **10** |
|  |  | **5** |
|  |  |  |
|  |  | **0** |

|  |  | ***** | **E** | **20** |  |  |  |
| --- | --- | --- | --- | --- | --- | --- | --- |
|  |  |  |  |  |  |  |  |
|  |  |  | **FastingGlucose(mmol/L)** | **15** |  |  |  |
|  |  |  |  |  |  |  |  |
|  |  |  |  | **5** |  |  |  |
|  |  |  |  |  |  |  |  |
|  |  |  |  |  |  |  |  |
|  |  |  |  | **10** |  |  |  |
|  |  |  |  | **0** |  |  |  |
| **IC** | | **A12** | |  | **IC** | | **A12** |
|  |  |  |  |  |  |  |  |

**Supplementary Figure 3:** Examination of the effect of cixutumumab (A12) a specific antibody leading to internalisation and degradation of the insulin like growth factor-1 receptor (IGF-1R) in high fat fed male C57BL/6J mice on plasma insulin, lipids compared to isotype control (IC) treated mice. **A)** Circulating concentrations of insulin (n=6), triglycerides (n=6), free fatty acids (n8-11) and leptin (n=8-11) were similar in cixutumumab and isotype control treated mice. **B)** Circulating levels of IGF-1 in cixutumumab treated mice tended to be higher in cixutumumab treated mice compared to IC treated mice (n=5). Data expressed as mean (SEM)* denotes P<0.05, *n* denotes number of mice per group, comparisons made using unpaired students t test.

| **A** |  |  |  |
| --- | --- | --- | --- |
| **PlasmaConcentration** | **20** | **PlasmaIGF-1Levels** | **(pg/ml)** |
|  | **15** |  |  |
|  |  |  |  |
|  | **10** |  |  |
|  | **5** |  |  |
|  | **0** |  |  |

|  |  |  |  |  |  | **l)** |  |  |  |  |  | **)** | |  |  | **)** |  |  |  |  |  | **l)** |
| --- | --- | --- | --- | --- | --- | --- | --- | --- | --- | --- | --- | --- | --- | --- | --- | --- | --- | --- | --- | --- | --- | --- |
|  |  |  |  |  |  | **/m** |  |  |  |  |  | **M** |  |  |  | **M** |  |  |  |  |  | **/m** |
|  |  |  |  |  | **g** | |  |  |  |  | **(m** | |  |  |  | **(m** |  |  |  |  |  | **g** |
|  |  |  |  | **p** | |  |  |  |  | **s** | |  |  |  |  | **s** |  |  |  |  | **n** | |
|  |  |  | **(** | |  |  |  |  |  |  |  |  |  |  |  |  |  |  |  | **(** | |  |
|  |  |  | **in** |  |  |  |  |  | **id** | |  |  |  |  |  | **id** |  |  |  | **n** |  |  |
|  |  |  |  |  |  |  |  |  | **r** |  |  |  |  |  | **c** | |  |  |  |  |  |  |
|  |  |  | **l** |  |  |  |  | **e** | |  |  |  |  |  |  |  |  |  | **ti** | |  |  |
|  |  | **u** | |  |  |  | **c** | |  |  |  |  |  | **y** | **A** |  |  | **p** | |  |  |  |
|  | **s** | |  |  |  | **y** | |  |  |  |  |  |  |  |  |  | **e** | |  |  |  |  |
| **n** | |  |  |  |  | **l** |  |  |  |  |  |  | **t** | |  | **L** | |  |  |  |  |  |
| **I** |  |  |  |  |  | **ig** |  |  |  |  |  |  | **t** | |  |  |  |  |  |  |  |  |
|  |  |  |  |  |  |  |  |  |  |  |  | **a** | |  |  |  |  |  |  |  |  |  |
|  |  |  |  |  |  | **r** |  |  |  |  |  |  |  |  |  |  |  |  |  |  |  |  |
|  |  |  |  |  |  | **T** |  |  |  |  |  | **eF** |  |  |  |  |  |  |  |  |  |  |
|  |  |  |  |  |  |  |  |  |  |  | **e** | |  |  |  |  |  |  |  |  |  |  |
|  |  |  |  |  |  |  |  |  |  | **r** | |  |  |  |  |  |  |  |  |  |  |  |
|  |  |  |  |  |  |  |  |  |  | **F** |  |  |  |  |  |  |  |  |  |  |  |  |

**B**

**1500**

**p=0.05**

**1000**

**500**

**0**

**IC**	**A12**

**Supplementary Figure 4: :** Examination of the effect of cixutumumab (A12) a specific antibody leading to internalisation and degradation of the insulin like growth factor-1 receptor (IGF-1R) in standard chow and

high fat fed male C57BL/6J mice: **A)** Brown adipose tissue expression of mRNA levels in Chow fed with isotype control (IC) and cixutumumab (A12)-fed mice (n=2-3) **B)** Epididymal fat pad expression of PPAR gamma mRNA levels in Chow fed with isotype control (IC) and cixutumumab (A12)-fed mice (n=2-3). Data expressed as mean (SEM). Comparisons made using unpaired students t test.

| **A** |  |  |  |  |  |  | **B** |  |  |
| --- | --- | --- | --- | --- | --- | --- | --- | --- | --- |
| **(Ratio)** | **10** | **IC** |  |  |  |  | **(Ratio)** | **5** |  |
|  |  | **A12** | |  |  |  |  |  |  |
|  | **8** |  |  |  |  |  |  | **4** |  |
|  |  |  |  |  |  |  |  |  |  |
| **Expression** | **6** |  |  |  |  |  | **Expression** | **3** |  |
|  | **4** |  |  |  |  |  |  | **2** |  |
|  |  |  |  |  |  |  |  |  |  |
| **Relative** | **2** |  |  |  |  |  | **Relative** | **1** |  |
|  |  |  |  |  |  |  |  |  |  |
|  | **0** |  |  |  |  | ***Tnf*** |  | **0** |  |
|  | *Ucp1* | *Vegf* | *Igf-1r* | *Leptin* | *Cidea* |  |  | **IC** | **A12** |

**Supplementary Figure 5: :** Examination of the effect of cixutumumab (A12) a specific antibody leading to internalisation and degradation of the insulin like growth factor-1 receptor (IGF-1R) in standard chow and high fat fed male C57BL/6J mice: **A)** Total FOXO expression in liver of High Fat fed with isotype control (IC) and cixutumumab (A12)-fed mice (n=5). **B)** FOXO phosphorylation levels in liver of High Fat fed with isotype control (IC) and cixutumumab (A12)-fed mice (n=5) **C)** FOXO phosphorylation levels in liver with IP injection of 0.75 units/kg insulin (Ins) in High Fat fed with isotype control (IC) and cixutumumab (A12)-fed mice (n=5). **D)** GSK expression in liver of High Fat fed with isotype control (IC) and cixutumumab (A12)-fed mice (n=5). **E)** GSK phosphorylation levels in liver of High Fat fed with isotype control (IC) and cixutumumab (A12)-fed mice (n=5). **F)** GSK phosphorylation levels in liver with injection of 0.75 units/kg insulin in High Fat fed with isotype control (IC) and cixutumumab (A12)-fed mice (n=5). FOXO-p / Relative FOXO expression was calculated as FOXO-p / (FOXO/Actin). GSK-p / Relative GSK expression was calculated as GSK-p / (GSK/Actin). Data expressed as mean (SEM). Representative blots presented on the far right. *n* denotes number of mice per group, comparisons made using unpaired students t test.

**A**

**FOXO / Actin Ratio**

**D**

| **1.5** | | **B** |  |  |  | **4** |  | **C** | **4** | **4** |  | **IC** |  | **A12** | **IC** |  | **A12** | **IC** | **A12** | **IC** |  | **A12** |
| --- | --- | --- | --- | --- | --- | --- | --- | --- | --- | --- | --- | --- | --- | --- | --- | --- | --- | --- | --- | --- | --- | --- |
|  |  | **Relative** | | **(*104** |  |  |  |  | **/Actin)*10** |  |  |  |  |  |  |  |  |  |  |  |  |  |
|  |  |  |  |  |  |  |  |  |  |  |  |  |  |  |  |  |  |  |  |  |  |  |
|  |  | **FOXO** | | **)** |  |  |  | **FOXO** | **)** |  | **71** | **C** |  | **C** | **Ins** | | **Ins** | **C** | **C** | **Ins** |  | **FOXO** |
|  |  |  | **(FOXO** | ***10** | |  |  |  | **(*10** |  |  |  |  |  |  |  |  |  |  |  |  |  |
|  |  |  | **Actin)** | **4** |  |  |  |  | **4** |  | **kDa** |  |  |  |  |  |  |  |  |  |  | **Ins** |
|  |  |  |  |  |  | **3** |  | **FOXO-p/Relative** |  | **3** | **71** |  |  |  |  |  |  |  |  |  |  | **FOXO-p** |
|  |  | **FOXO** | | **Expression** |  |  |  |  | **ExpressionFOXO-/(FOXO** |  |  |  |  |  |  |  |  |  |  |  |  |  |
| **1.0** | |  | **/** |  |  |  |  |  |  |  |  |  |  |  |  |  |  |  |  |  |  |  |
|  |  |  |  |  |  |  |  |  |  |  |  |  |  |  |  |  |  |  |  |  |  |  |
|  |  |  |  |  |  |  |  |  |  |  |  |  |  |  |  |  |  |  |  |  |  |  |
|  |  |  |  |  |  | **2** |  |  |  | **2** |  |  |  |  |  |  |  |  |  |  |  |  |
| **0.5** | | **-p /-p/** | |  |  | **1** |  |  |  | **1** |  |  |  |  |  |  |  |  |  |  |  |  |
|  |  |  | **FOXO** |  |  |  |  |  |  |  | **45** |  |  |  |  |  |  |  |  |  |  | **Actin** |
|  |  |  |  |  |  |  |  |  |  |  |  |  |  |  |  |  |  |  |  |  |  |  |
|  |  |  |  |  |  |  |  |  |  |  |  |  |  |  |  |  |  |  |  |  |  |  |
| **0.0** | |  |  |  |  | **0** |  |  |  | **0** |  |  |  |  |  |  |  |  |  |  |  |  |
|  | **Iso C** | **A12 C** |  |  |  |  | **Iso C** | **A12 C** |  | **Iso C Ins** | **A12 Ins** |  |  |  |  |  |  |  |  |  |  |  |
|  | **3** | **E** |  |  | **1.5** | |  | **F** |  | **3** |  |  |  |  |  |  |  |  |  |  |  |  |
|  |  |  |  |  |  |  |  |  | **4** |  |  |  |  |  |  |  |  |  |  |  |  |  |
|  |  |  |  |  |  |  |  |  |  |  |  | **Isotype** | | | | **A12** | | **Isotype** | | | **A12** | |
| **GSK/ActinRatio** |  | **GSKSK** | **Expression*10** | |  |  |  | **GSK-p/RelativeGSK** | **ExpressionGSK-/(GSK/Actin)*10** |  |  |  |  |  |  |  |  |  |  |  |  |  |
|  |  |  |  |  |  |  |  |  |  |  |  |  |  |  |  |  |  |  |  |  |  |  |
|  |  | **GSK** | **4** | |  |  |  |  | **)** |  |  |  |  |  |  |  |  |  |  |  |  |  |
|  |  | **(GSK/Actin)** | **(*10** | |  |  |  |  | **4** |  |  |  |  |  |  |  |  |  |  |  |  |  |
|  |  |  | **)** | |  |  |  |  | **(*10** |  | **kDa** |  | **C** | **Ins** | | **C** | **Ins** | **C** | **Ins** | **C** | | **Ins** |
|  | **2** | **Relative** | **4** | | **1.0** | |  |  |  | **2** |  |  |  |  |  |  |  |  |  |  |  |  |
|  |  |  |  |  |  |  |  |  |  |  |  |  |  |  |  |  |  |  |  |  |  |  |
|  |  |  |  |  |  |  |  |  |  |  |  |  |  |  |  |  |  |  |  |  |  |  |
|  |  |  |  |  |  |  |  |  |  |  | **51** |  |  |  |  |  |  |  |  |  |  | **GSK-p** |
|  |  |  |  |  |  |  |  |  |  |  |  |  |  |  |  |  |  |  |  |  |  |  |
|  | **1** | **/** |  |  | **0.5** | |  |  |  | **1** |  |  |  |  |  |  |  |  |  |  |  | **GSK** |
|  |  | **-p** |  |  |  |  |  |  |  |  |  |  |  |  |  |  |  |  |  |  |  |  |
|  |  | **/** |  |  |  |  |  |  |  |  |  |  |  |  |  |  |  |  |  |  |  |  |
|  |  | **-p** |  |  |  |  |  |  |  |  | **51** |  |  |  |  |  |  |  |  |  |  |  |
|  |  |  |  |  |  |  |  |  |  |  | **45** |  |  |  |  |  |  |  |  |  |  | **Actin** |
|  | **0** |  |  |  | **0.0** | |  |  |  | **0** |  |  |  |  |  |  |  |  |  |  |  |  |
|  |  |  |  |  |  |  |  |  |  |  |  |  |  |  |  |  |  |  |  |  |  |  |
|  | **Iso C** | **A12 C** |  |  |  |  | **Iso C** | **A12 C** |  | **Iso C Ins** | **A12 Ins** |  |  |  |  |  |  |  |  |  |  |  |

**Supplementary Figure 6: Liver blots. :** Examination of the effect of cixutumumab (A12) a specific antibody leading to internalisation and degradation of the insulin like growth factor-1 receptor (IGF-1R) in standard chow and high fat fed male C57BL/6J mice. Akt-Ser473, Akt, Actin blots with IP injection of 0.75 units/kg insulin (Ins) in High Fat fed with isotype control (IC) and cixutumumab (A12)-fed mice (n=5).

**Supplementary Figure 7: Muscle blots :** Examination of the effect of cixutumumab (A12) a specific antibody leading to internalisation and degradation of the insulin like growth factor-1 receptor (IGF-1R) in standard chow and high fat fed male C57BL/6J mice. Akt-Ser473, Akt, Actin blots with IP injection of 0.75 units/kg insulin (Ins) in High Fat fed with isotype control (IC) and cixutumumab (A12)-fed mice (n=5).
