# Supplementary material for: Cixutumumab reveals a critical role for IGF-1 in adipose and hepatic tissue remodelling during the development of diet-induced obesity
Source: Adipocyte. 2022 Jun 23;11(1):366–78. doi: 10.1080/21623945.2022.2089394 (PMC9235901; doi:10.1080/21623945.2022.2089394)
Supplement: Supplemental Material [file KADI_A_2089394_SM2687.zip › A12Supplementary.doc]

**Supplementary Figure 1:** Examination of the effect of cixutumumab (A12) a specific antibody leading to internalisation and degradation of the insulin like growth factor-1 receptor (IGF-1R) in standard chow and

high fat fed male C57BL/6J mice: **A)** Treatment schedule. Mice were fed standard chow or high fat diet for 6-weeks and received 10mg/Kg cixutumumab or isotype control (IC) every 3 days by intraperitoneal injection for

a 3-week period from 3 weeks onwards. **B)** Cixutumumab treated chow fat fed mice had a reduction in IGF-1R expression in liver, fat and muscle (*n*=7-9). **C)** No effect of cixutumumab on insulin receptor (IR) expression in liver, epididymal fat, and muscle in chow-fed mice (*n*=7-9). **D)** Cixutumumab treated high-fat fed mice had a reduction of IGF-1R expression in liver, epididymal fat and muscle (*n*=7-9). **E)** No effect of cixutumumab on IR expression in liver, epididymal fat or muscle in high-fat fed mice (*n*=7-9). Data expressed as mean (SEM)* denotes P<0.05, *n* denotes number of mice per group, comparisons made using unpaired students t test).

**A**

| **High-fat/chow** | GTT/ITT |  |  |  | **A12** |  |  |  | GTT/ITT | Organharvest |
| --- | --- | --- | --- | --- | --- | --- | --- | --- | --- | --- |
|  |  |  |  |  |  |  |  |  |  |  |
|  |  | 10mg/kg **A12** or **Isotype control (IC)** | | | | | | |  |  |
| Day 0 |  |  |  |  |  |  |  |  |  |  |
|  |  |  |  |  |  |  |  |  |  |  |
|  | 21 | 23 | | 27 | 30 | 34 | 37 |  | 41 |  |

**C**	**D**

**5**

| **/Actin** |  | **4** |
| --- | --- | --- |
| **Expression** | **(Ratio)** |  |
|  |  | **3** |
|  |  |  |
|  |  | **2** |
| **1R** |  | **1** |
| **IGF-** |  |  |
|  |  |  |
|  |  | **0** |

| **B** |  | **2.5** |  |  |  | **IC** |  |  | **Liver** |  |  |
| --- | --- | --- | --- | --- | --- | --- | --- | --- | --- | --- | --- |
|  |  |  |  |  |  |  |  |  |  |  |  |
| **/Actin** |  | **2.0** |  |  |  | **A12** | **C** | **C** | **HF** |  | **HF** |
|  |  |  |  |  |  |  |  |  |  |  |  |
|  |  |  |  |  |  |  |  |  |  |  |  |
| **expression** | **(Ratio)** | **1.5** |  |  |  |  | **A12** | **IC** | **A12** |  | **IC** |
|  |  |  |  |  |  |  |  |  |  |  | **IGF1-Rb** |
|  |  |  |  |  |  |  |  |  |  |  |  |
|  |  |  |  |  |  |  |  |  |  |  |  |
|  |  | **1.0** |  |  | ***** |  |  |  |  |  | **IRb** |
| **1R** |  |  |  |  |  | ***** |  |  |  |  |  |
|  |  |  | ***** |  |  |  |  |  |  |  |  |
|  |  | **0.5** |  |  |  |  |  |  |  |  |  |
| **IGF-** |  |  |  |  |  |  |  |  |  |  | **b-actin** |
|  |  |  |  |  |  |  |  |  |  |  |  |
|  |  | **0.0** |  |  |  |  |  |  |  |  |  |
|  |  |  | **Liver** | **Fat** | | **Muscle** |  |  |  |  |  |
|  |  |  |  | **E** |  |  |  | **Fat** | | **Muscle** | |
|  |  |  |  |  | **3** |  |  |  |  |  |  |
|  |  |  | **IC** |  |  |  | **IC** |  |  |  |  |
|  |  |  |  |  |  |  |  |  |  |  |  |
|  |  |  | **A12** |  |  |  | **A12** | **HF** | **HF** | **HF** | **HF** |
|  |  |  |  | **/ Actin** |  |  |  |  |  |  |  |
|  |  |  |  |  |  |  |  |  |  |  |  |
| ***** |  |  |  |  | **2** |  |  | **IC** | **A12** | **IC** | **A12** |
|  |  |  |  | **Expression** | **(Ratio)** |  |  |  |  |  | **IRb** |
|  |  |  |  |  |  |  |  |  |  |  | **IGF-1Rb** |
|  | ***** |  |  |  | **1** |  |  |  |  |  |  |
|  |  |  |  |  |  |  |  |  |  |  |  |
|  |  |  | ***** | **IR** |  |  |  |  |  |  |  |
|  |  |  |  |  |  |  |  |  |  |  | **b actin** |
|  |  |  |  |  | **0** |  |  |  |  |  |  |
|  |  |  |  |  |  |  |  |  |  |  |  |

**Liver**	**Fat**	**Muscle**	**Liver**	**Fat**	**Muscle**

**Supplementary Figure 2:** Examination of the effect of cixutumumab (A12) a specific antibody leading to internalisation and degradation of the insulin like growth factor-1 receptor (IGF-1R) in high fat fed male C57BL/6J mice on plasma insulin, lipids compared to isotype control (IC) treated mice. **A)** Circulating concentrations of insulin, triglycerides, free fatty acids and leptin were similar in cixutumumab and isotype control treated mice. **B)** Circulating levels of IGF-1 in cixutumumab treated mice tended to be higher in cixutumumab treated mice compared to IC treated mice. Data expressed as mean (SEM)* denotes P<0.05, *n* denotes number of mice per group, comparisons made using unpaired students t test.

| **A** |  |  |
| --- | --- | --- |
| **PlasmaConcentration** | **20** | **PlasmaIGF-1Levels** |
|  | **15** |  |
|  |  |  |
|  | **10** |  |
|  | **5** |  |
|  | **0** |  |

|  |  |  |  |  |  | **l)** |  |  |  |  | **)** | |  |  | **)** |  |  |  |  |  | **l)** |
| --- | --- | --- | --- | --- | --- | --- | --- | --- | --- | --- | --- | --- | --- | --- | --- | --- | --- | --- | --- | --- | --- |
|  |  |  |  |  |  | **/m** |  |  |  |  | **M** |  |  |  | **M** |  |  |  |  |  | **/m** |
|  |  |  |  |  | **g** | |  |  |  | **(m** | |  |  |  | **(m** |  |  |  |  |  | **g** |
|  |  |  |  | **p** | |  |  |  | **s** | |  |  |  |  | **s** |  |  |  |  | **n** | |
|  |  |  | **(** | |  |  |  |  |  |  |  |  |  |  |  |  |  |  | **(** | |  |
|  |  |  | **in** |  |  |  |  | **id** | |  |  |  |  |  | **id** |  |  |  | **n** |  |  |
|  |  |  |  |  |  |  |  | **r** |  |  |  |  |  | **c** | |  |  |  |  |  |  |
|  |  |  | **l** |  |  |  | **e** | |  |  |  |  |  |  |  |  |  | **ti** | |  |  |
|  |  | **u** | |  |  | **c** | |  |  |  |  |  | **y** | **A** |  |  | **p** | |  |  |  |
|  | **s** | |  |  |  | **ly** |  |  |  |  |  |  |  |  |  | **e** | |  |  |  |  |
| **n** | |  |  |  |  |  |  |  |  |  |  | **t** | |  | **L** | |  |  |  |  |  |
| **I** |  |  |  |  |  | **ig** |  |  |  |  |  | **t** | |  |  |  |  |  |  |  |  |
|  |  |  |  |  |  |  |  |  |  |  | **a** | |  |  |  |  |  |  |  |  |  |
|  |  |  |  |  |  | **r** |  |  |  |  |  |  |  |  |  |  |  |  |  |  |  |
|  |  |  |  |  |  | **T** |  |  |  |  | **eF** |  |  |  |  |  |  |  |  |  |  |
|  |  |  |  |  |  |  |  |  |  | **e** | |  |  |  |  |  |  |  |  |  |  |
|  |  |  |  |  |  |  |  |  | **r** | |  |  |  |  |  |  |  |  |  |  |  |
|  |  |  |  |  |  |  |  |  | **F** |  |  |  |  |  |  |  |  |  |  |  |  |

**B**

**1500**

**p=0.05**

| **(pg/ml)** | **1000** |
| --- | --- |
|  | **500** |
|  |  |
|  | **0** |

**IC**	**A12**
